# LPIN1 promotes triglycerides synthesis and is transcriptionally regulated by PPARG in buffalo mammary epithelial cells

**DOI:** 10.1038/s41598-022-06114-w

**Published:** 2022-02-11

**Authors:** Fangting Zhou, Xinyang Fan, Yongwang Miao

**Affiliations:** 1 Faculty of Animal Science and Technology, Yunnan Agricultural University, Kunming, 650201 Yunnan China; 2 College of Chemistry, Biology and Environment, Yuxi Normal University, Yuxi, 653100 Yunnan China

**Keywords:** Cell biology, Molecular biology, Physiology

## Abstract

Studies on 3T3-L1 cells and HepG2 hepatocytes have shown that phosphatidic acid phosphohydrolase1 (LPIN1) plays a key role in adipogenesis, acting as a co-activator of peroxisome proliferator-activated receptor gamma coactivator 1a (PGC-1a) to regulate fatty acid metabolism. However, the functional role and regulatory mechanism of *LPIN1* gene in milk fat synthesis of buffalo are still unknown. In this study, overexpression of buffalo *LPIN1* gene transfected with recombinant fusion expression vector significantly increased the expression of *AGPAT6*, *DGAT1*, *DGAT2*, *GPAM* and *BTN1A1* genes involved in triglyceride (TAG) synthesis and secretion, as well as *PPARG* and *SREBF1* genes regulating fatty acid metabolism in the buffalo mammary epithelial cells (BMECs), while the lentivirus-mediated knockdown of buffalo *LPIN1* dramatically decreased the relative mRNA abundance of these genes. Correspondingly, total cellular TAG content in the BMECs increased significantly after *LPIN1* overexpression, but decreased significantly after *LPIN1* knockdown. In addition, the overexpression or knockdown of *PPARG* also enhanced or reduced the expression of *LPIN1* and the transcriptional activity of its promoter. The core region of buffalo *LPIN1* promoter spans from − 666 bp to + 42 bp, and two PPAR response elements (PPREs: PPRE1 and PPRE2) were identified in this region. Site mutagenesis analysis showed that PPARG directly regulated the transcription of buffalo *LPIN1* by binding to the PPRE1 and PPRE2 on its core promoter. The results here reveal that the *LPIN1* gene is involved in the milk fat synthesis of BMECs, and one of the important pathways is to participate in this process through direct transcriptional regulation of PPARG, which in turn significantly affects the content of TAG in BMECs.

## Introduction

Phosphatidic acid phosphohydrolase1 (LPIN1), as a member of the lipid protein family, was identified during positional cloning of the mutant gene underlying lipodystrophy in the fatty liver dystrophy (fld) mouse^1^. In mammals, there are three subtypes of LPIN: LPIN1, LPIN2 and LPIN3. They have the same structural characteristics, including at least one nuclear localization signal domain (NLS), one amino-terminal domain (N-LIP) and one carboxyl-terminal domain (C-LIP)^2^. The N-LIP and C-LIP are highly conserved in different mammals. All the members of LPIN not only play the role of phosphatidic acid phosphorylase (PAP), which are necessary for TAG synthesis, but also act as transcriptional co-activators to regulate the expression of genes related to the lipid synthesis^3^. Among the three LPINs, the PAP specific activity of LPIN1 was significantly higher than that of LPIN2 and LPIN3. In humans and mice, *LPIN1* is highly expressed in adipose tissue, skeletal muscle, myocardium and testis, but low in liver, kidney, brain and other tissues^4^. In mouse liver and 3T3-L1 cells, LPIN1 deficiency can prevent normal lipid accumulation and induce expression of key adipogenic genes, including peroxisome proliferator-activated receptor (PPAR) and CCAAT enhancer-binding protein (C/EBP)^5^. In addition, studies have shown that *LPIN1* gene can be induced to express in the late stage of 3T3-L1 preadipocyte differentiation^6^. After *LPIN1* is interfered by siRNA, the 3T3-L1 cells cannot differentiate into mature adipocytes. On the contrary, the overexpression of *LPIN1* gene can promote their differentiation and maturation^6^. These results reveal that LPIN1 plays an important role in adipocyte maturation and adipogenesis. Previous studies also showed that *LPIN1* is the most highly expressed subtype of the LPIN family, and its expression level on the 60th day of lactation is 15 times higher than that on the 15th day before lactation, suggesting that LPIN1 may be involved in milk fat synthesis^7^. The *LPIN1* is also a downstream gene of the PPARG signaling pathway in the mammary gland of dairy cows^8^. In yak, the expression level of *LPIN1* during the peak lactation is higher than that during other lactation and dry period, further suggesting that this gene may be an important gene related to lactation process^9^.

Peroxisome proliferator-activated receptor gamma (*PPARG*) is a critical gene that regulates lipid metabolism, body immunity, insulin resistance and adipocyte differentiation^10^. In 3T3-L1 cells, overexpression of *LPIN1* increases the expression of *PPARG* and the accumulation of lipid droplets, while knockdown of *LPIN1* gene could inhibit the adipogenesis induced by PPARG agonists and also reduce phosphorylation levels of PPARG and mitogen-activated protein kinase (ERK1/2)^11^. The study by Kim et al^12^ also demonstrated that in the absence of ligand rosiglitazone, LPIN1 activated PPARG by releasing nuclear receptor corepressor 1 (NCOR1), retinoic acid and thyroid hormone receptor silencing mediator (SMRT). The physical interaction between LPIN1 and PPARG occurs in the LPIN1 C-terminal region^12^. These results suggest that LPIN1 has dual functions of transcriptional co-factor and phosphatidic acid phosphorylase. In fatty acid metabolism, PPARG can bind to retinoid-X-receptor (RXR) to regulate the expression of downstream genes^13^. PPAR/RXR is activated by ligands, and then binds to specific DNA sequences (PPAR response element, PPRE) in the promoter region of specific target genes, regulates gene transcription, and induces lipid metabolism and cell differentiation^13^. In the goat mammary epithelial cells, the *PPARG* gene can regulate the transcription of adipose differentiation-related protein (*ADRP*) and stearoyl-CoA desaturase 1 (*SCD*) genes by binding to PPRE of their promoter to promote milk fat synthesis^14,15^.

Although some functional roles of *LPIN1* gene in dairy cow lactation have been clarified, the transcriptional regulation mechanism of *LPIN1* gene remains unclear. Buffalo and cattle belong to different genera, and the solid content of buffalo milk is significantly higher than that of cattle milk, especially its milk fat content is almost twice that of cow milk^16^, suggesting that the genetic basis of lactation may be different between buffalo and dairy cows. So far, the specific role of *LPIN1* gene in buffalo milk fat synthesis and its transcriptional regulation are still unknown. In this study, we hypothesized that LPIN1 plays an important role in the milk fat synthesis of buffalo mammary epithelial cells (BMECs), and that PPARG can regulate its transcription by binding to the potential PPREs in the promoter region of the buffalo *LPIN1* gene. In order to confirm this hypothesis, *LPIN1* knockdown by lentivirus-mediated interference and overexpression by transfecting recombinant fusion expression vector were performed in BMECs. Furthermore, we cloned the promoter of buffalo *LPIN1* gene, and studied the transcriptional regulation mechanism of buffalo *LPIN1* gene through the search of PPREs and the site-directed mutagenesis of this element, as well as the overexpression and interference of the *PPARG* gene. This study will provide strong evidence to clarify the role of LPIN1 in the synthesis of buffalo milk fat.

## Results

### Identification of the BMECs

**Figure 1:**
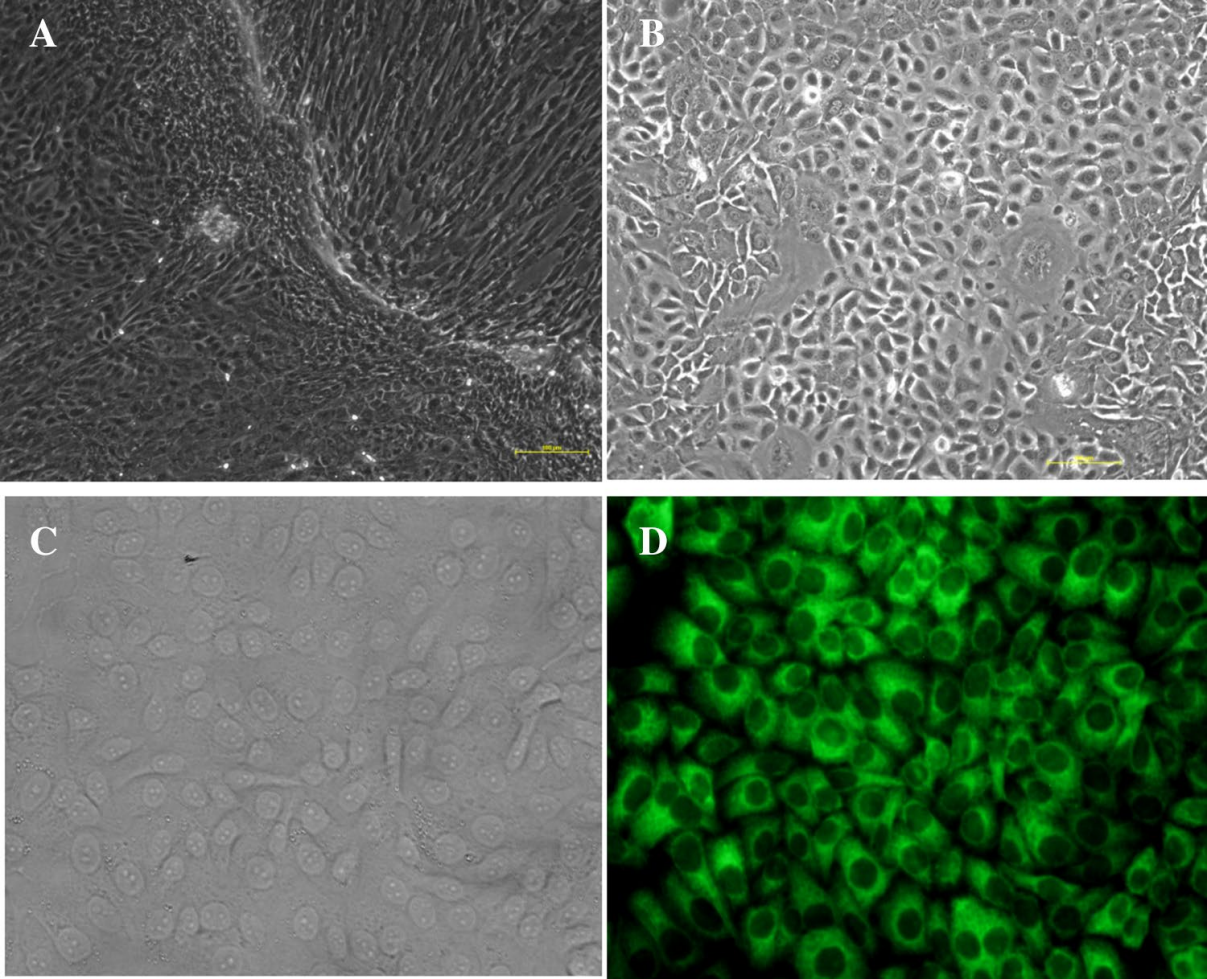
Photomicrographs of isolation, culture and identification of BMECs. (**A**) Mixed population of epithelial and fibroblast cells; (**B**) The BMECs purified three times; (**C**) Bright light image of BMECs stained with cytokeratin 18 (400 ×); (**D**) Fluorescent image of BMECs stained with cytokeratin 18 (400 ×).

The BMECs were isolated by tissue block culture, which grew into a mixed population of cobblestone epithelial cells surrounded by spindle-shaped fibroblasts around them (Fig. 1A). After the mixed cells were purified three times with 0.25% trypsin EDTA, the fully purified island and cobblestone-like epithelial cells were obtained (Fig. 1B). Immunocytochemical detection showed that cytokeratin 18 were expressed in the epithelial cells (Fig. 1C, D), which revealed that the cells we obtained were BMECs.

### Construction of LPIN1 overexpression vector and its effect on gene expression in the BMECs

**Figure 2:**
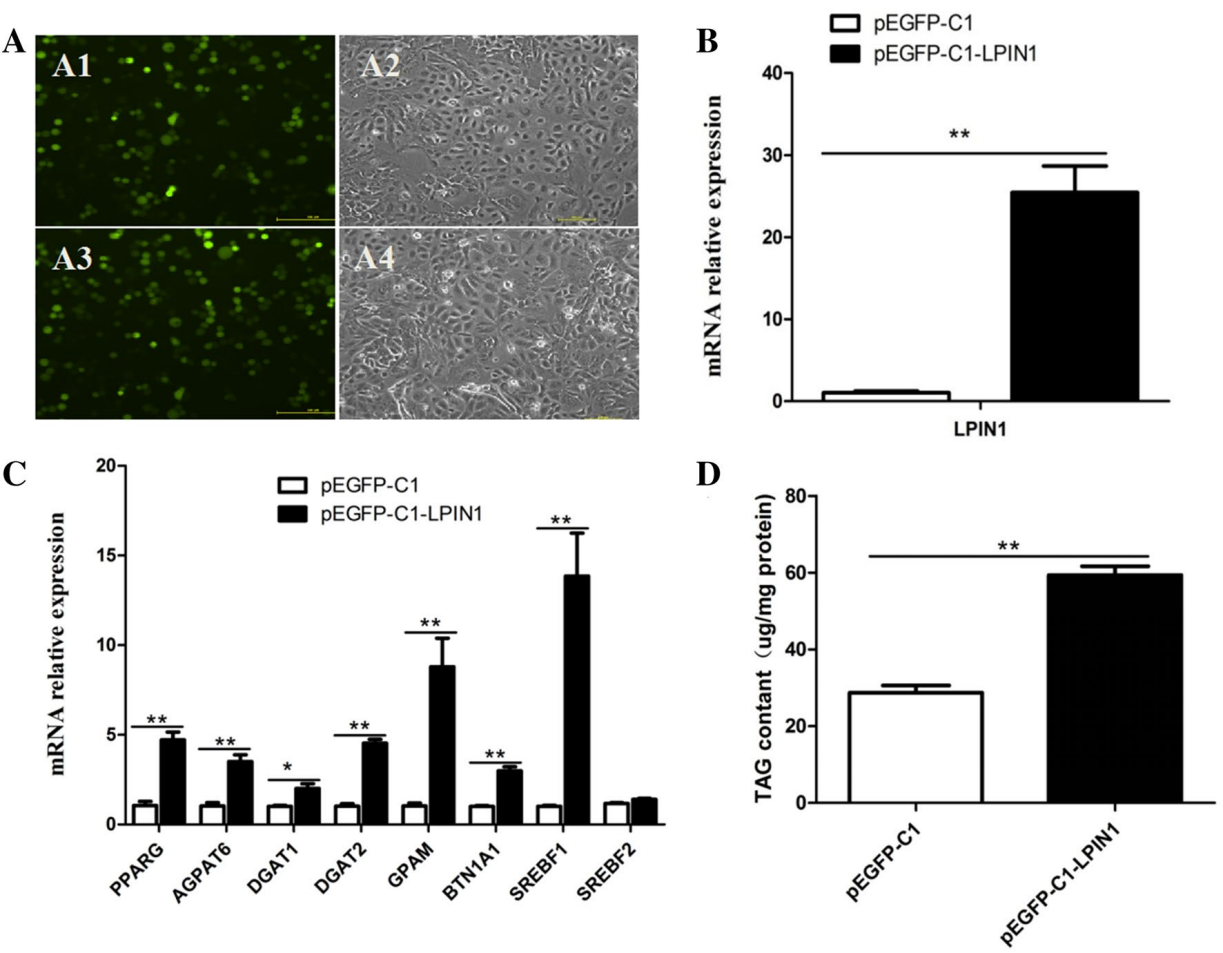
Effect of *LPIN1* overexpression in the BMECs. (**A)** Fluorescence results of *LPIN1* overexpression in the BMECs. (**A1**). Green fluorescence of control group (pEGFP-C1); (**A2**) Ordinary light of control group (pEGFP-C1); A3. Green fluorescence of pEGFP-C1-LPIN1; A4. Ordinary light of pEGFP-C1-LPIN1; (**B**) The efficiency of *LPIN1* overexpression; (**C**) The overexpression of *LPIN1* up-regulates the expression of genes related to milk fat synthesis in the BMECs; (**D)** Changes of total TAG content in the BMECs under the *LPIN1* overexpression. Values are displayed as means ± SEM for three biological replicates. *, *P* < 0.05; **, *P* < 0.01.

The CDS of *LPIN1* gene was first ligated to pEASY-T1 Simple Cloning vector, and then ligated to the pEGFP-C1 vector. The pEGFP-C1-LPIN1 and pEGFP-C1 were directly transfected into the BMECs, respectively. After 48 h, green fluorescence was observed under fluorescence inverted microscope (Leica, DMI4000B, Germany), which suggested that the pEGFP-C1-LPIN1 was successfully transfected into the BMECs (Fig. 2A). Compared with the control (pEGFP-C1), the mRNA abundance of *LPIN1* in the BMECs transfected with pEGFP-C1-LPIN1 was significantly increased (25 fold) (*P* < 0.01; Fig. 2B). Meanwhile, the overexpression of *LPIN1* significantly increased mRNA abundance of *AGPAT6* (~ 3.4 fold), *DGAT1* (~ 2.0 fold), *DGAT2* (~ 4.4 fold), *GPAM* (~ 8.5 fold) and *BTN1A1* (~ 2.9 fold) genes that involved in TAG synthesis (Fig. 2C). In addition, the overexpression of *LPIN1* gene also significantly increased the expression of *PPARG* (~ 4.5 fold) and *SREBF1* (~ 13.5 fold) genes related to the regulation of fatty acid metabolism, but had no significant effect on the expression of *SREBF2* (*P* > 0.05; Fig. 2C).

In order to determine whether the expression of *LPIN1* affects the accumulation of TAG in the BMECs, the intracellular TAG content was measured using the TAG kit, and the results showed that the content of TAG in the pEGFP-C1-LPIN1 group was significantly higher than that in the pEGFP-C1 group (*P* < 0.01; Fig. 2D).

### Construction of LPIN1 interference vector and its effect on gene expression in the BMECs

**Figure 3:**
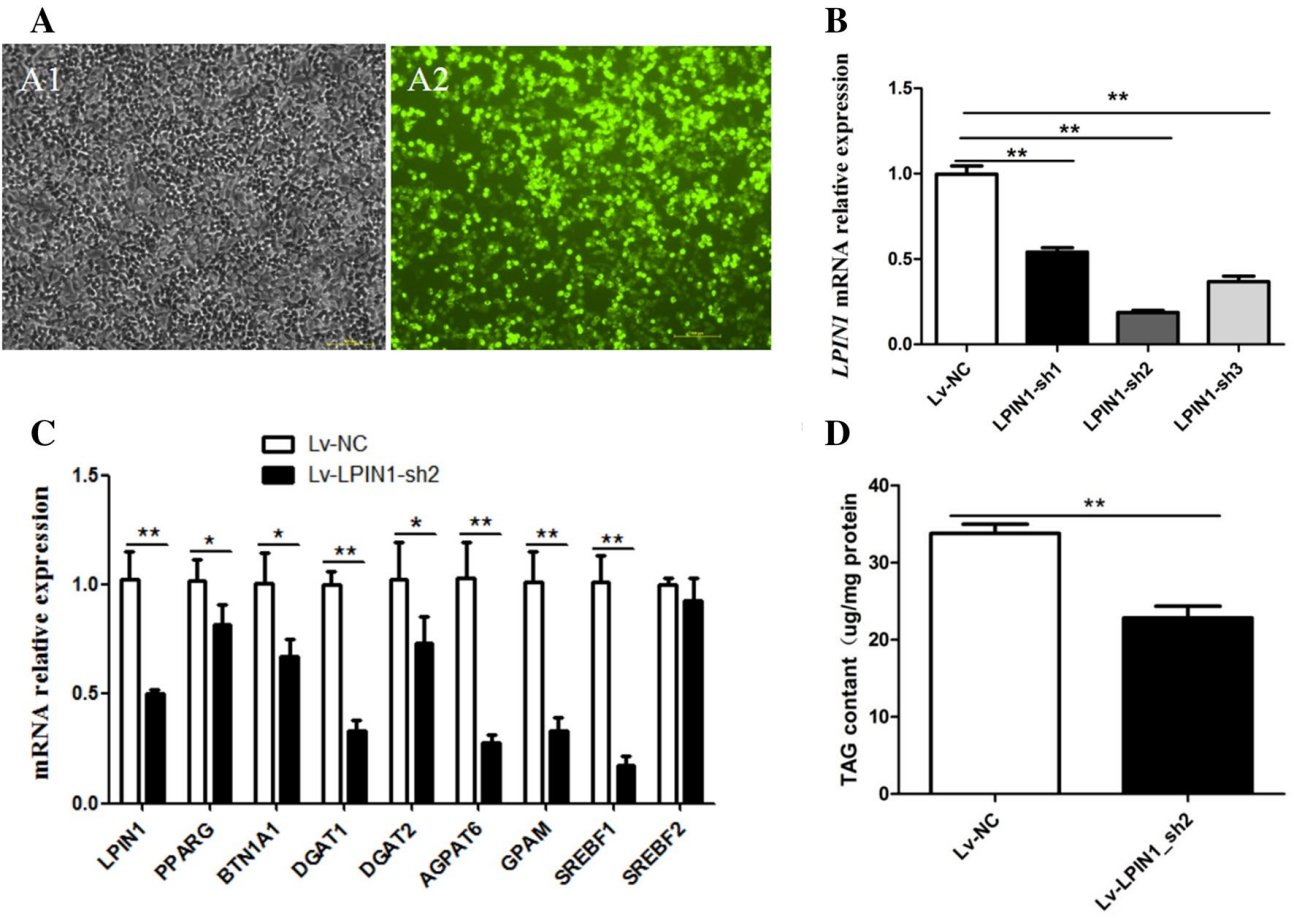
Effect of *LPIN1* interference in the BMECs. (**A**) The image of green fluorescence to identify transfection efficiency of shRNAs in HEK-293-T cells; A1. The image of ordinary light of co-transfected with pLKO.1-shRNA and pEGFP-C1-LPIN1; A2. The image of green fluorescence of co-transfected with pLKO.1-shRNA and pEGFP-C1-LPIN1; (**B**) *LPIN1* interference efficiency of three shRNA recombinants evaluated via RT-qPCR; (**C**) Interference of *LPIN1* down-regulates the expression of genes related to milk fat synthesis in the BMECs; (**D**) The effects of *LPIN1* interference on the total cellular TAG content in the BMECs. Values are expressed as means ± SEM for three biological replicates. *P < 0.05; **P < 0.01.

Three pairs of shRNAs were ligated into the PLKO.1 vector to construct recombinant plasmids (pLKO.1-shRNAs) for interfering with *LPIN1* gene. The pLKO.1-shRNA and pEGFP-C1-LPIN1 vectors were co-transfected into HEK-293 T cell, and the green fluorescence results showed that the transfection efficiency could meet the experimental requirements (Fig. 3A). Compared with pEGFP-C1-LPIN1 group, the expression level of *LPIN1* in the cells co-transfected with pEGFP-C1-LPIN1 and LPIN1-sh1, LPIN1-sh2, LPIN1-sh3 decreased by 46%, 82% and 63%, respectively. It shows that LPIN1-sh2 has the highest efficiency of *LPIN1* knockdown (Fig. 3B).

The LPIN1-sh2 was selected to prepare lentivirus particles (Lv-LPIN1-sh2) and further transfected into BMECs. The results showed that the mRNA abundance of *LPIN1* in the Lv-LPIN1-sh2 group was significantly lower than that in control (50%) (*P* < 0.01; Fig. 3C). The knockdown of *LPIN1* significantly decreased the mRNA abundance of genes related to milk fat synthesis in the BMECs (Fig. 3C), and the relative expression of *AGPAT6*, *DGAT1*, *DGAT2*, *GPAM* and *BTN1A1* genes involved in TAG synthesis decreased by 73%, 67%, 29%, 68% and 33%, respectively. Meanwhile, the knockdown of *LPIN1* also decreased the transcriptional levels of genes involved in regulating fatty acid metabolism. For example, the expression of *PPARG* and *SREBF1* was reduced by 22% and 83%, respectively, but it had no significant effect on the expression of *SREBF2* (Fig. 3C).

Correspondingly, compared with the control group, the TAG content of the BMECs in the Lv-LPIN1-sh2 group was significantly reduced (*P* < 0.01) (Fig. 3D).

### Characterization and activity analysis of buffalo LPIN1 promoter

**Figure 4:**
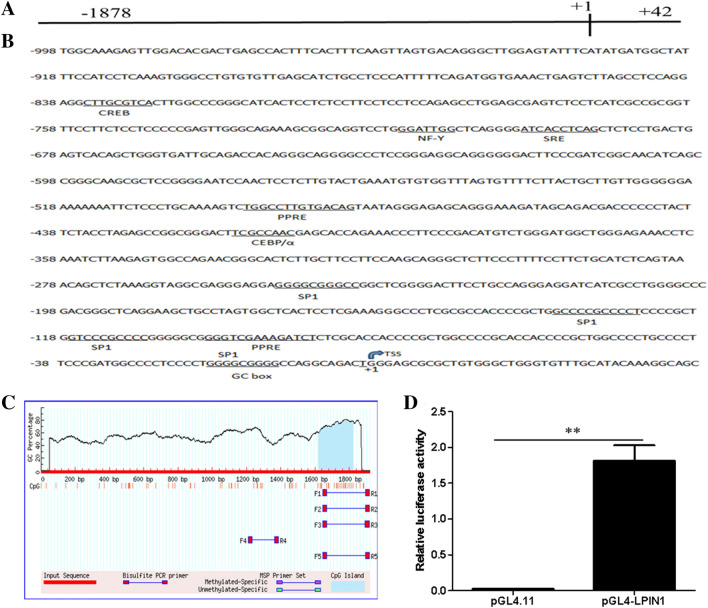
Sequence identification and activity analysis of buffalo *LPIN1* promoter. (**A**) Fragment length of the amplified *LPIN1* promoter; (**B**) Analysis of regulatory elements in the promoter sequence of *LPIN1* gene; (**C**) The prediction of CpG island; (**D**) The luciferase activity of *LPIN1* promoter. **, *P* < 0.01.

We obtained a sequence of 1920 bp at the 5’ end of the buffalo *LPIN1* gene by PCR, which contains 1878 bp upstream of the transcription start site (TSS) (+ 1) (Fig. 4A). Bioinformatics analysis showed that the G + C content of the amplified sequence was as high as 56.67%, containing a GC box (− 11 bp to − 19 bp), but not a typical TATA box. The sequence also contained several important cis-acting elements: PPAR response elements (PPRE1: − 481 bp to − 493 bp; PPRE2: − 86 bp to − 100 bp), sterol regulatory element (SRE: − 691 bp to − 700 bp) and the sites of nuclear factor (NF-Y: − 709 bp to − 715 bp), C/EBPα (− 408 bp to − 425 bp) and cAMP-response element binding protein (CREB: − 827 bp to − 835 bp) (Fig. 4B). The analysis by Methprimer software showed that the amplified sequence also contains a CpG island (Fig. 4C).

The pGL4-LPIN1 luciferase recombinant vector containing *LPIN1* gene promoter (− 1878 bp to + 42 bp) was constructed, then transfected into the BMECs to determine the luciferase activity. Compared with the control (pGL4.11), the luciferase activity in the BMECs in the pGL4-LPIN1 group was significantly increased (~ 62 fold), indicating that the − 1878 to + 42 region of *LPIN1* gene contains the promoter region of this gene (Fig. 4D; *P* < 0.01).

### Identification of the core promoter region of LPIN1 gene

**Figure 5:**
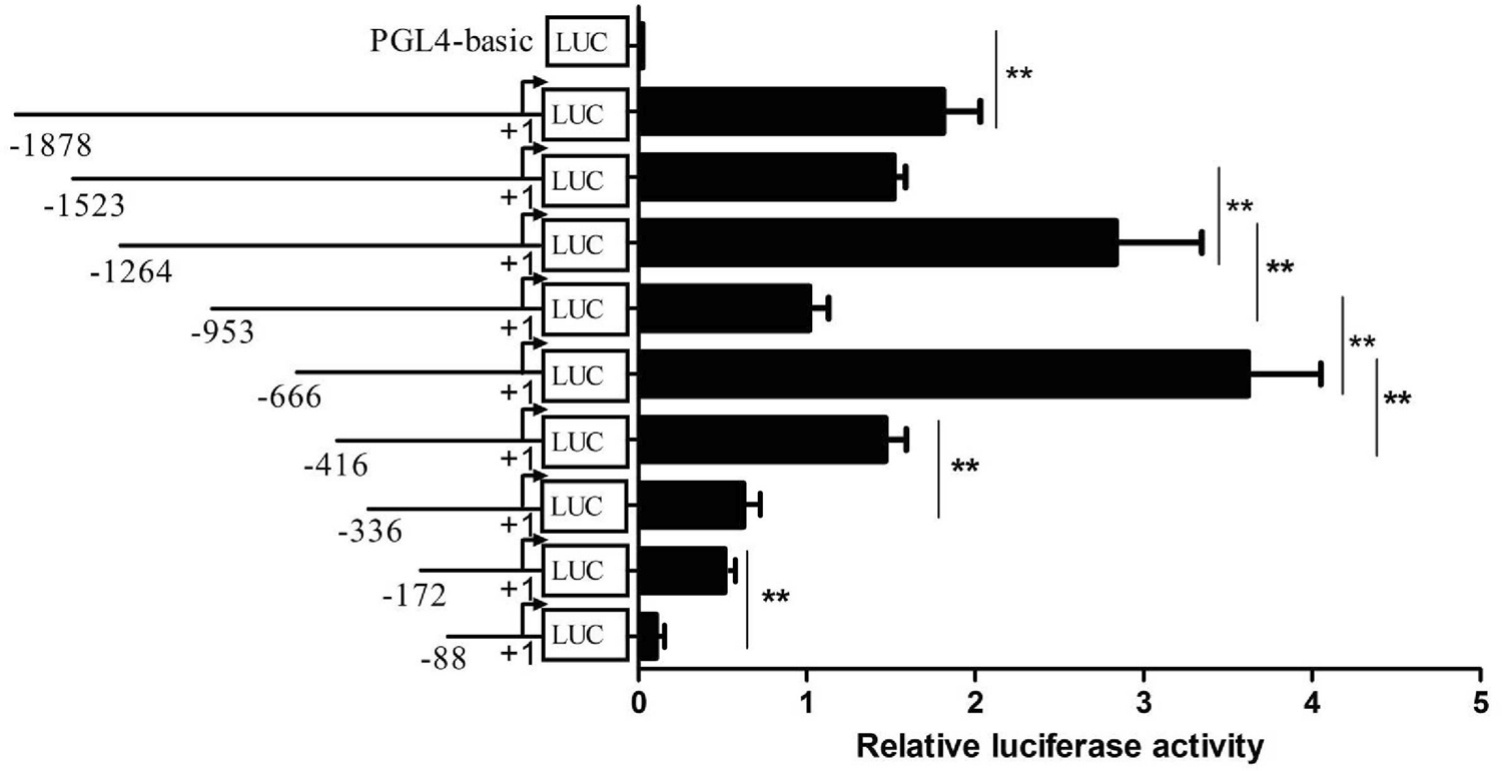
Luciferase activity of 5’-flanking fragment vectors of buffalo *LPIN1* promoter in the BMECs. Relative luciferase activity (Firefly: Renilla) after 48 h transfection with different 5’-flanking deletion constructs (− 1878/ + 42 bp, − 1523/ + 42 bp, − 1264/ + 42 bp, − 953/ + 42 bp, − 666/ + 42 bp, − 416/ + 42 bp, − 336/ + 42 bp, − 172/ + 42 bp and − 88/ + 42 bp). **, *P* < 0.01.

A series of luciferase reporter recombinant vectors with 5’ flanking deletion fragments of the *LPIN1* promoter were constructed (− 1523/ + 42, − 1264/ + 42, − 953/ + 42, − 666/ + 42, − 416/ + 42, − 336/ + 42, − 172/ + 42 and − 88/ + 42). After transfecting these recombinant vectors into BMECs, luciferase activity was detected (Fig. 5). The deletion from − 1878 bp to − 1523 bp showed a decrease of luciferase activity that was trending toward significance (*P* = 0.07); when the deletion reached the − 1264 bp, the luciferase activity was significantly increased (*P* < 0.01); When the deletion reached − 953 bp, the luciferase activity was significantly reduced (*P* < 0.01), indicating that there are negative regulatory elements in the region from − 1264 bp to − 953 bp. In addition, the deletion from − 953 bp to − 666 bp has the highest luciferase activity (*P* < 0.01), and the deletion from − 666 bp to − 416 bp reduced the luciferase activity by about 56% (*P* < 0.01). Subsequently, with the deletion of the 5’ flanking region, the activity continued to decline (Fig. 5). This indicates that the fragment between − 666 bp and + 42 bp contains cis-functional elements required for transcriptional activation of *LPIN1* gene. The above results reveal that the core region of the *LPIN1* promoter is located in the region from − 666 bp to + 42 bp, which has basic transcriptional activity.

### Overexpression of PPARG increased the activity of LPIN1 promoter

**Figure 6:**
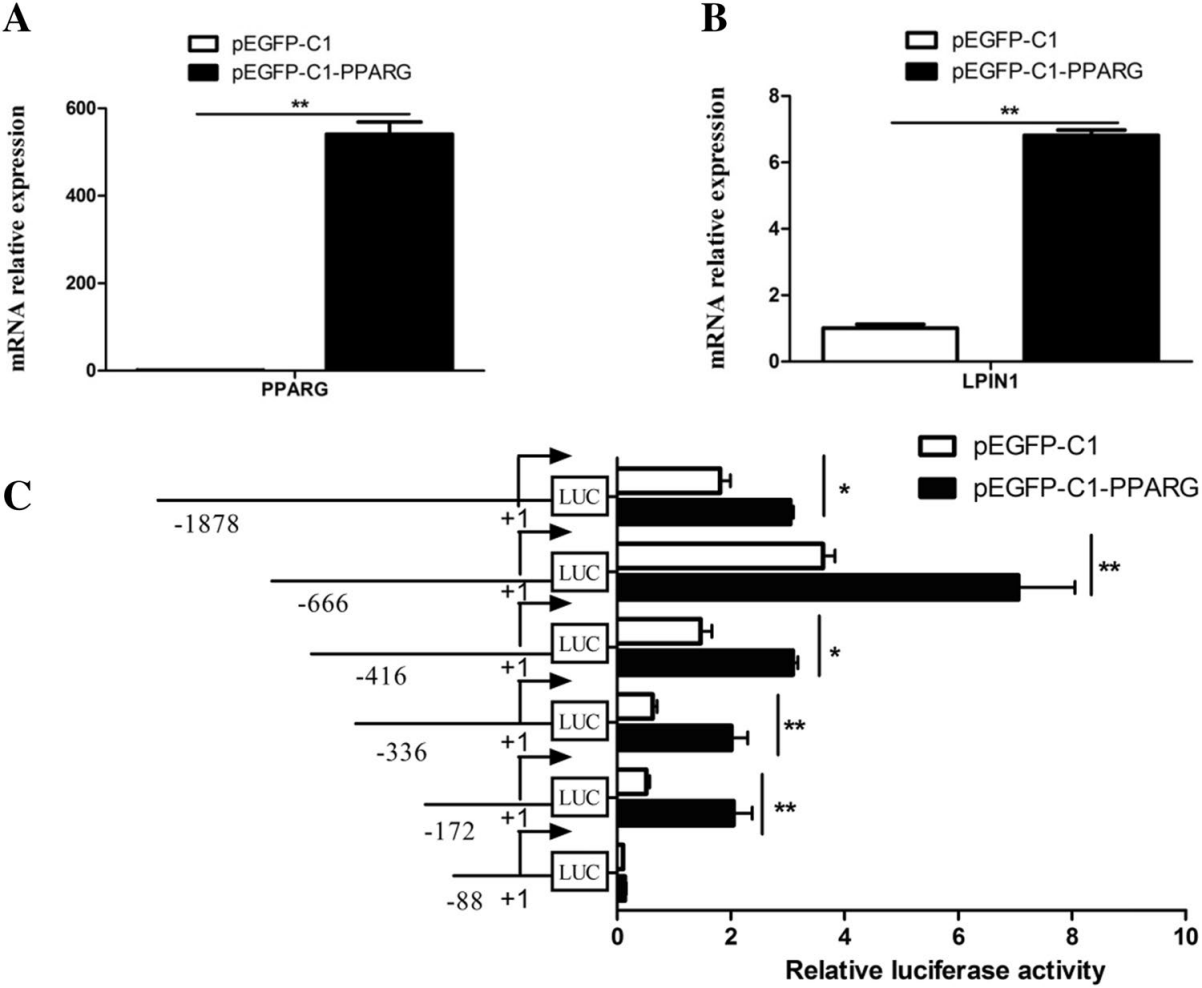
The effects of *PPARG* overexpression on the *LPIN1* mRNA expression and promoter activity in the BMECs. (**A**) The mRNA expression of *PPARG* after transfection with pEGFP-C1-PPARG; (**B**) The mRNA expression of *LPIN1* after transfection with pEGFP-C1-PPARG; (**C**) Relative luciferase activity (Firefly: Renilla) after 48 h transfection of pEGFP-C1-PPARG and different 5’ flanking deletion constructs (− 1878 bp/ + 42 bp, − 666/ + 42 bp, − 416/ + 42 bp, − 336/ + 42 bp, − 172/ + 42 bp and − 88/ + 42 bp. Values are provided as mean ± SEM; *P < 0.05; **P < 0.01.

In order to determine whether PPARG has a regulatory effect on the *LPIN1* gene promoter, the pGL4-LPIN1 vector or 5’ deletion mutagenesis constructs were transfected with pEGFP-C1-PPARG to detect their luciferase activity. The results showed *PPARG* gene was successfully overexpressed in the BMECs, and its expression level was 540 times that of the control (pEGFP-C1) (Fig. 6A). In addition, the overexpression of *PPARG* significantly increased the mRNA expression of *LPIN1* gene (~ 6.8 fold; Fig. 6B). Compared with the control (pEGFP-C1), after overexpression of *PPARG* (pEGFP-C1-PPARG), the activity of the 5’ deletion mutagenesis constructs was significantly increased except for the deletion fragment − 88/ + 42 (Fig. 6C). Therefore, it is speculated that there are PPRE elements in the *LPIN1* promoter, and PPARG may regulate the expression of the *LPIN1* gene by combining with the PPRE elements.

### Interference of PPARG decreased the activity of LPIN1 promoter

**Figure 7:**
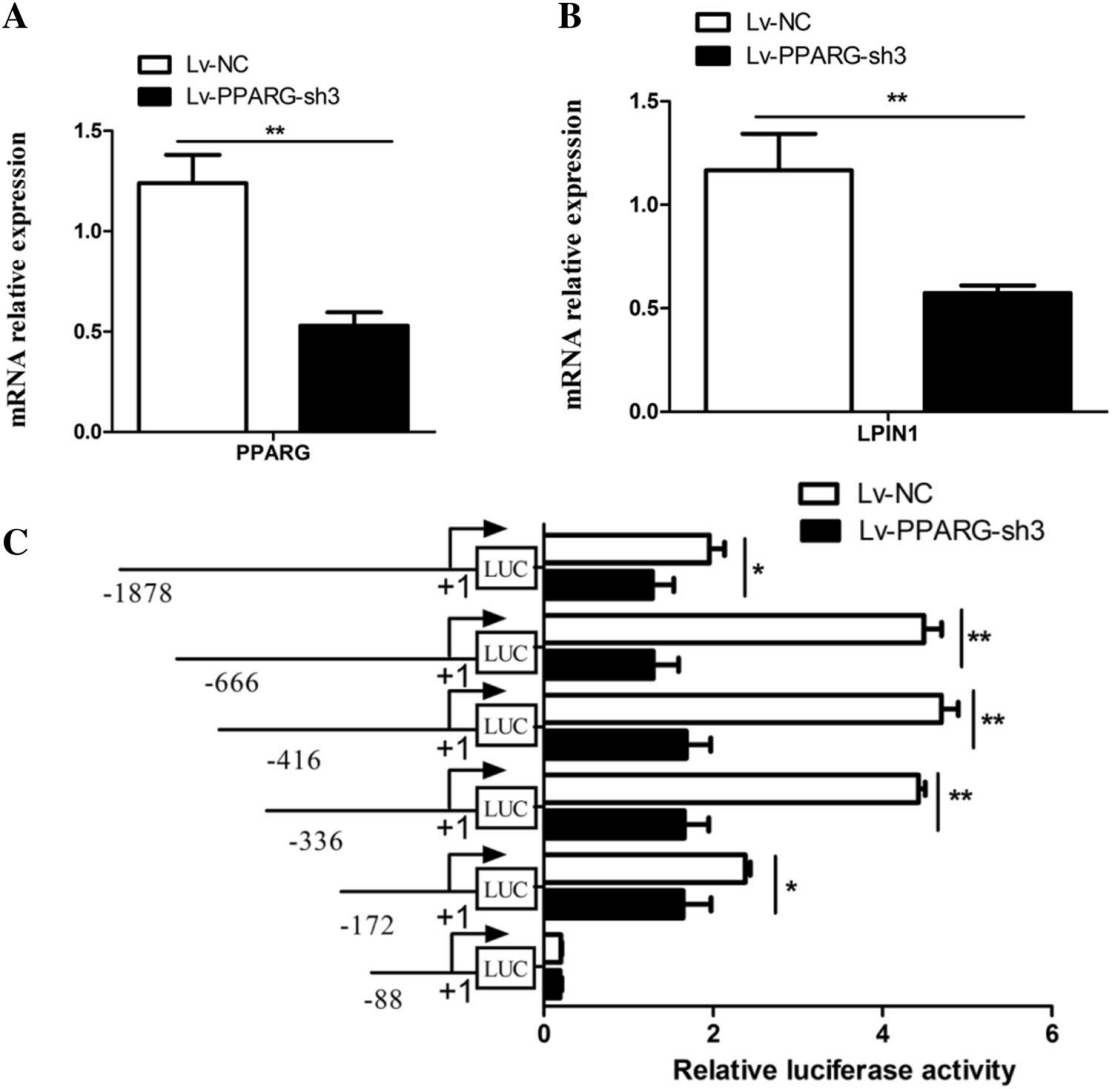
Effects of *PPARG* knockdown on *LPIN1* promoter activity in the BMECs. (**A**) The mRNA expression of *PPARG* after treatment with Lv-PPARG-sh3; (**B**) The mRNA expression of *LPIN1* after treatment with Lv-PPARG-sh3; (**C**) Relative luciferase activity (Firefly: Renilla) of 5’ flanking deletion constructs (− 1878 bp/ + 42 bp, − 666/ + 42 bp, − 416/ + 42 bp, − 336/ + 42 bp, − 172/ + 42 bp and − 88/ + 42 bp). Values are provided as mean ± SEM; *, *P* < 0.05; **, *P* < 0.01.

After the shRNA mediated-knockdown of *PPARG* was conducted in BMECs, the mRNA expression of *LPIN1* and *PPARG* and the luciferase activity of each promoter fragment were measured. The results showed that after interference with the *PPARG* gene (Lv-PPARG-sh3), the mRNA level of this gene was significantly reduced (*P* < 0.01; Fig. 7A), and the mRNA level of the *LPIN1* gene also decreased significantly (*P* < 0.01; 43%; Fig. 7B). In addition, compared with the control (Lv-NC), after interfering with PPARG, except for that of the − 88 bp/ + 42 bp fragment vector, the luciferase activity of all the 5’ deletion fragment vectors decreased significantly (Fig. 7C). This result further confirms that there are PPARG binding target sites (PPREs) in the *LPIN1* promoter, and PPARG may directly regulate its expression by binding to the PPREs on the promoter of *LPIN1* gene.

### Site-directed mutation of the PPRE decreased basal activity of the LPIN1 promoter

**Figure 8:**
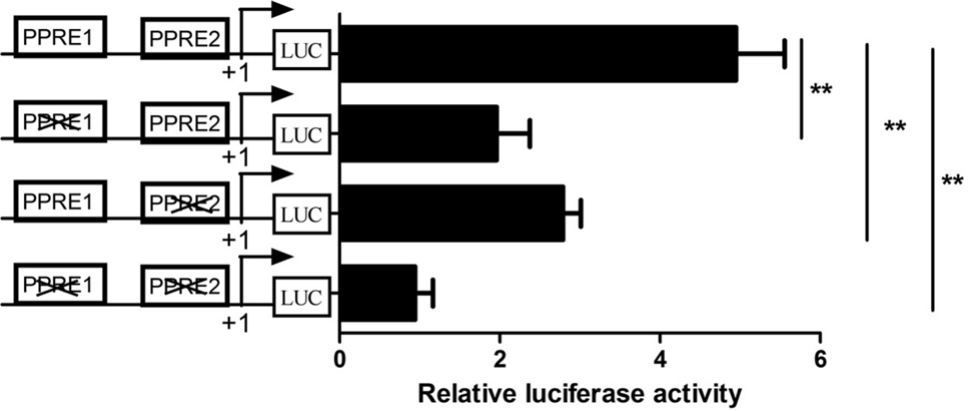
Site-directed mutagenesis of PPREs in the *LPIN1* promoter reduces its transcriptional activity regulated by PPARG. **, *P* < 0.01.

To determine whether PPARG regulates the expression of the gene by binding to the two predicted PPREs (PPRE1 and PPRE2) of the *LPIN1* promoter, recombinant vectors with the site-directed mutations on the PPREs located in the core promoter region of *LPIN1* (− 666/ + 42 bp) were constructed, respectively, including the recombinants for site-directed mutagenesis of PPRE1 and PPRE2, and vector for simultaneous mutagenesis of both. Luciferase detection showed that both PPRE1 and PPRE2 site-directed mutagenesis could significantly decrease the promoter activity of *LPIN1* gene. The decrease of LPIN1 promoter activity after site-directed mutation of PPRE1 (62%) was greater than that after site-directed mutation of PPRE2 (44%) (Fig. 8). In addition, after the simultaneous site-directed mutation of PPRE1 and PPRE2, the *LPIN1* promoter activity decreased to a greater extent compared to PPRE1 or PPRE2 mutation alone (82%) (Fig. 8). It is suggested that PPARG can directly regulate the transcription of *LPIN1* gene by binding to the PPRE1 and PPRE2, and the PPRE1 plays a more important role in the binding of PPARG.

## Discussion

The mammary gland of lactating cows is a formidable TAG synthesis machine^7^. The synthesis of milk fat in the mammary gland includes biological processes such as uptake and transport of long-chain fatty acids, de novo synthesis of fatty acids, desaturation, synthesis of triglycerides, formation and desaturation of lipid droplets. Milk fat synthesis is regulated by PPARG, SREBF and other transcription factors^17^. The lipid droplets formed in the endoplasmic reticulum of BMECs are released into the acinar cavity of the mammary gland in the form of milk fat globules^18^. In the TAG synthesis pathway, glycerol-3-phosphate acyltransferase (GPAM) first converts glycerol-3-phosphate (G-3-P) and fatty acyl-CoA into a lysophosphatidic acid (LPA) in the endoplasmic reticulum. Then, LPA is converted into a phosphatidic acid (PA) by the AGPAT enzyme. PA is subsequently dephosphorylated by the enzyme LPIN1 to form a diacylglycerol (DAG). Then, DGAT makes DAG plus fatty acid acyl-CoA to form a TAG, which is the rate-limiting enzyme for TAG synthesis^19^. As a member of the immunoglobulin superfamily, butyrophilin A1 (BTN1A1) is essential for regulating the secretion of milk fat droplets^20^. In this study, the functional role of LPIN1 in buffalo milk fat synthesis was investigated by lentivirus-mediated interference and overexpression experiments in *vitro*. The overexpression of *LPIN1* gene led to a significant increase in mRNA expression of *GPAM*, *DGAT1*, *DGAT2* and *BTN1A1* genes involved in TAG synthesis and secretion in the BMECs, as well as a significant increase in TAG content (Fig. 2). On the contrary, after the knockdown of *LPIN1* gene, the expression of these genes in the BMECs was decreased, and the TAG content was also significantly decreased (Fig. 3). This suggests that buffalo *LPIN1* gene plays an important role in the triglyceride pathway of buffalo milk fat synthesis, and it is speculated that this gene also participates in the synthesis of TAG in BMECs by catalyzing the dephosphorylation of PA to form DAG. Previous studies in goat mammary epithelial cells showed that knockdown of *PPARG* gene led to a significant decrease in *LPIN1* gene, suggesting that *LPIN1* gene may be a downstream target gene of PPARG during milk fat synthesis in ruminants^14^. Our previous research revealed that PPARG is a central regulator of buffalo milk fat synthesis^21^. Another study found that LPIN1, as a transcription co-activator, its C-terminal amino acid residues at 217–399 can enhance the transcriptional activation activity of PPARG^12^. In this study, the expression of *PPARG* gene was significantly increased after the overexpression of *LPIN1* gene (Fig. 2). Moreover, the overexpression of *PPARG* gene also significantly increased the expression of *LPIN1* gene (Fig. 6). Therefore, it was speculated that in the BMECs, the *LPIN1* gene involved in milk fat synthesis may be regulated by the PPARG. Meanwhile, LPIN1 may also activate PPARG through transcriptional co-activation, and then PPARG regulates the milk fat synthesis of BMECs, which needs further confirmation in subsequent experiments.

SREBF1 is considered to be an important central regulator in the regulatory network of milk fat synthesis^22,23^. Previous studies have shown that *SREBF1* gene is a target gene of PPARG^24^. Based on the fact that the expression of *SREBF1* gene was significantly down-regulated after *LPIN1* interference in this study (Fig. 3), it is speculated that the interference of *LPIN1* gene initially reduced the expression of *PPARG*, which further led to the down-regulation of *SREBF1* expression. The main function of SREBF2 is to specifically regulate cholesterol synthesis^22^. In this study, the overexpression or interference of *LPIN1* gene had no significant effect on the expression of *SREBF2*, suggesting that *LPIN1* gene may not be involved in the synthesis of cholesterol in BMECs.

Study has shown that the expression of *LPIN1* gene is regulated by cholesterol, and SREBF1 can directly bind to the sterol regulatory element (SRE) on the *LPIN1* promoter to promote the transcription of this gene^25^. In humans and mice, *LPIN1* is regulated by a variety of transcription factors, including SREBF1^26^, estrogen-related receptor γ (ERRγ)^27^, hepatic nuclear factor 4a (HNF4a)^28^ and C/EBPα^6^. They directly bind to the corresponding sites in the *LPIN1* promoter to promote *LPIN1* transcription and lipid synthesis. In this study, not only the binding sites of SREBF1 and C/EBPα were found in the promoter of the buffalo *LPIN1*, but also the PPREs sites. In the fatty acid synthesis pathway, PPARG binds to RXR to form a heterodimer, which then binds to the ligand and enters the nucleus, and then recognizes the PPREs on the target gene promoter, thus activating the transcription of downstream genes^13,29^. We found two PPREs (PPRE1: TGGCCTTGTGACAG; PPRE2: GGGTCGAAAGATCT) in the promoter of buffalo *LPIN1*, which were highly consistent with the recognized PPRE^13^_._ So far, PPRE has been found in the promoter of many genes related to lipid synthesis, such as thrombospondin receptor (CD36)^30^, adipose differentiation-related protein (ADRP) and stearoyl-CoA desaturase 1 (SCD)^15^. In this study, after the overexpression of *PPARG*, the luciferase activity of all recombinants with 5’ deletion fragments of the *LPIN1* promoter increased significantly except for fragment − 88/ + 42 (Fig. 6). In contrast, after interference with *PPARG*, the luciferase activity of all recombinants with 5’ deletion fragments of the *LPIN1* promoter was significantly decreased except for fragment − 88/ + 42 (Fig. 7). Furthermore, the site-directed mutagenesis of PPRE1 and PPRE2 in the core promoter of *LPIN1* greatly reduced the transcriptional activity of the promoter fragment, and after the simultaneous site-directed mutagenesis of PPRE1 and PPRE2, the activity of the core promoter was significantly lower than that of PPRE1 and PPRE2 alone (Fig. 8). The results here indicate that PPARG can directly regulate the transcription of buffalo *LPIN1* by binding to the PPRE1 and PPRE2 sites on its promoter.

## Materials and methods

### Ethics declarations

Sample collection in the described experiments was approved by the Animal Care and Use Committee of Yunnan Agricultural University (No. YNAU2019llwyh019). Every effort has been made to minimize suffering. All methods were performed in accordance with the relevant guidelines and regulations and the study is reported in accordance with ARRIVE guidelines.

### Isolation, culture and identification of buffalo mammary epithelial cells

BMECs were isolated, purified and cultured from buffalo mammary gland tissue at the peak of lactation using the previously described method^21^. The female buffalo used for sampling (Binglangjiang buffalo, an indigenous river type buffalo breed distributed in western Yunnan Province, China) was healthy, five years old and at the 3rd parity (about 60 d postpartum). In short, the fresh buffalo mammary tissue was collected and washed with the phosphate buffer saline (PBS, Gibco, USA) for three times. Part of the acini was washed by highly resistant PBS (containing 400 IU mL-1 penicillin and 400 IU mL-1 streptomycin). The tissue blocks were then cut into 1 to 2 mm^3^ pieces and spread at the petri dishes, then cultured in an incubator with the condition of 37 °C, 5% CO_2_ and 95% air humidity. The culture medium is DMEM/F12 (Gibco, USA) supplemented with fetal bovine serum (10%, Gibco, USA), penicillin/streptomycin (10 kU/L, Gibco, USA), bovine insulin (5 mg/L, Sigma, USA), hydrocortisone (5 mg/L, Sigma, USA), epidermal growth factor (1 mg/L, Sigma, USA) and 5 μg/mL holotransferrin (Sigma, USA). The cells began to grow about 12 days. The cells were digested with 0.25% trypsin-ethylenediamine tetraacetic acid (EDTA, Gibco, USA) for 2–3 min, and the exfoliated fibroblasts were then washed with the PBS. The remaining cells adhered to the bottom of the petri dish were further digested for 5–10 min and centrifuged at 1250 rpm for 3 min. The purified cells were collected for subculture. After 3 times of purification, the purified BMECs could be obtained. The identification protocol of BMECs is as follows: When the purified BMECs were confluent with 70–80%, the cells were washed with 1 × PBS, fixed with 4% paraformaldehyde for 20 min, then incubated 30 min with 1 × BSTX (1 × PBS containing 10% BSA, 0.1% Triton X-100) (Sigma, USA) for blocking. The cells were then incubated with 1:100 diluted cytokeratin 18 antibody (Sigma, USA) for 1 h, then were washed for three times and incubated with FITC-labeled monoclonal anti-mouse IgG secondary antibody (Sigma, USA) at 1:100 dilutions for 1 h. In the end, the purified cells were taken images with a microscope (Leica, DMI4000B, Germany).

### Construction of vector for overexpression and interference of LPIN1 gene

Based on the mRNA sequence of buffalo *LPIN1* (XM_006073743), a pair of primers were designed using Primer Premier 5.0 software^31^: LPIN1-sense, 5’-AAGCTTATGAACTACGTCGGGCAG-3’ (Hind III restriction endonuclease site was underlined); LPIN1-antisense, 5’-GGTACCCTAGGCTGATGCAGAATGAAC-3’ (KpnI restriction endonuclease site was underlined). The PCR product was purified by a Gel Extraction Kit (OMEGA, USA), and then cloned into pEASY-T1 Simple Cloning vector (TransGen, China). Subsequently, the target fragment was ligated into the pEGFP-C1 (Takara, China) vector to construct an overexpression recombinant vector, referred as pEGFP-C1-LPIN1. Then, the positive recombinants were purified using the EndoFree Maxi Plasmid Kit (QIAGEN, Germany) for subsequent cell transfection experiment. The sequencing verification was completed by Shanghai Biological Engineering Technology Services Co., Ltd. (Shanghai, China).

According to the mRNA sequence of buffalo *LPIN1* gene (XM_006073743), three pairs of short hairpin RNAs (shRNAs) targeting the *LPIN1* gene were designed using the online software BLOCK-iT RNAi Designer (http://rnaidesigner.invitrogen.com/rnaiexpress/). After the shRNA sequences were annealed, it was ligated into the pLKO.1 vector to generate final recombinant plasmids (pLKO.1-shRNAs). All the recombinant vectors were sequenced for verification and then purified using EndoFree Maxi Plasmid Kit (QIAGEN, Germany). The sequences of shRNAs are presented in Table 1.

**Table 1:** Information of shRNAs used for the knockdown of *LPIN1*.

Name of shRNA	Sequences (5’-3’)
LPIN1_sh1_F	CCGGGCTGGACAGCAGTAGAATTCTCTCGAGAGAATTCTACTGCTGTCCAGCTTTTTG
LPIN1_sh1_R	AATTCAAAAAGCTGGACAGCAGTAGAATTCTCTCGAGAGAATTCTACTGCTGTCCAGC
LPIN1_sh2_F	CCGGGCTCCTGGAGAGAATAATTTGCTCGAGCAAATTATTCTCTCCAGGAGCTTTTTG
LPIN1_sh2_R	AATTCAAAAAGCTCCTGGAGAGAATAATTTGCTCGAGCAAATTATTCTCTCCAGGAGC
LPIN1_sh3_F	CCGGGGCGGTCTTTCTAGCTCTTGCCTCGAGGCAAGAGCTAGAAAGACCGCCTTTTTG
LPIN1_sh3_R	AATTCAAAAAGGCGGTCTTTCTAGCTCTTGCCTCGAGGCAAGAGCTAGAAAGACCGCC

### Overexpression of buffalo LPIN1 gene

When the confluence of BMECs in a 6-well plate is 70–80%, pEGFP-C1-LPIN1 was transfected into the BMECs using Lipo6000™ transfection reagent (Beyotime Biotechnology, China) in accordance with the manufacturer’s protocol, and pEGFP-C1 was transfected as negative control. Forty-eight hours after transfection, the green fluorescent protein (GFP) was monitored using a Leica fluorescent microscope (DMI4000B, Germany). Then, the BMECs were harvested for total RNA extraction or triacylglycerol (TAG) content analysis.

### Knockdown of buffalo LPIN1 gene

HEK-293 T cells (purchased from Kunming Institute of Zoology, Chinese Academy of Sciences) were cultured in DMEM/F12 medium containing 10% fetal bovine serum and 1% penicillin/streptomycin (10 kU/L, Gibco, USA). HEK-293 T cells with a confluence rate of 70–80% were co-transfected with pLKO.1-shRNA and pEGFP-C1-LPIN1 at a ratio of 3:1 to screen for the most effective shRNA targeting the *LPIN1* gene. The pEGFP-C1-LPIN1 vector was transfected alone as a control. Forty-eight hours after transfection, the expression of GFP was monitored using a Leica fluorescent microscope (DMI4000B, Germany). The HEK-293 T cells were then collected for RT-qPCR analysis. The PLKO.1 system used for the RNAi was a gift from David Root Lab^32^. When HEK-293 T cells were cultured to 70–80% confluence in a 10 cm plate, Lipo6000 (Beyotime Biotechnology, China) was used to co-transfection pLKO.1-shRNA, pMD2G (enveloped plasmid) and pPAX2 (packaged plasmid) into HEK-293 T cells at a ratio of 5:3:2 to produce lentivirus (Lv-LPIN1-shRNA). In addition, pLKO.1, pMD2G and psPAX2 were co-transfected into HEK-293 T cells as a negative control group (Lv-NC). The culture medium contained the lentivirus was centrifuged by 400 g for 5 min at 4 °C by a high-speed freezing centrifuge (Sorvall ST8; Thermo Fisher Scientific, USA) and then filtered with a 0.45 μm filter. The lentivirus particle was stored at − 80 °C for long-term storage.

When the BMECs were cultured in a 60 mm plate to 70–80% confluence, the lentivirus (Lv-LPIN1-shRNA or Lv-NC) was added to culture medium. Meanwhile, 2 mL of polybrene (2 μg/mL, Sigma, USA) was added to the culture medium to improve the efficiency of virus infection. The medium was replaced with fresh medium after 24 h. The BMECs were harvested after 48 h for RNA extraction or TAG content analysis. Since PLKO.1 vector does not express fluorescent protein, multiplicity of infection (MOI) was not estimated in this study, and only RT-qPCR was used to ensure sufficient interference efficiency.

### Gene expression detection

Total RNA was extracted and purified from the BMECs, and the cDNA was synthesized using a PrimeScript RT kit (TaKaRa, China). RT-qPCR primer pairs spanning exon-exon were designed with Primer Premier 5.0 to avoid amplification of genomic DNA. Detailed information about the primer pairs is present in Table 2. The qPCR was performed an Applied Biosystems™ 7500 (Thermo Fisher, USA) with TB Green® Advantage® qPCR Premix (Takara, China). The efficiency of amplification was determined using program LinRegPCR (https://www.medischebiologie.nl/files/?main=files&fileName=LinRegPCR.zip&description=LinRegPCR:%20qPCR%20data%20analysis&sub=LinRegPCR). The ubiquitously expressed genes of *ACTB* (actin beta), *GAPDH* (glyceraldehyde-3-phosphate dehydrogenase) and *YWHAZ* (tyrosine 3-monooxygenase tryptophan 5-monooxygenase activation protein zeta) were used as internal reference genes to normalize the target gene expression^21^. The genes for expression detection here included the *PPARG*, glycerol-3-phosphate acyltransferase (*GPAM*), diacylglycerol acyltransferase 1 (*DGAT1*), diacylglycerol acyltransferase 2 (*DGAT2*), 1-acylglycerol-3-phosphate O-acyltransferases6 (*AGPAT6*), lipid phosphate phosphohydrolase1 (*LPIN1*), butyrophilin A1 (*BTN1A1*), sterol regulatory element-binding transcription factor 1 (*SREBF1*) and sterol regulatory element-binding transcription factor 2 (*SREBF2*). They are associated with the synthesis and regulation of milk fat^33^. The mRNA abundance of these genes was quantified using the 2^-ΔΔCt^ method. The ΔCt = Ct (target genes)-Ct (reference genes). ΔΔCt = ΔCt-ΔCt (control). The control in the formula was group Lv-NC in the knockdown experiment, and pEGFP-C1 in gene overexpression experiment. Finally, the *ACTB*, *GAPDH* and *YWHAZ* were used as internal reference genes to calculate the 2^-ΔΔCt^ of each gene, and then the geometric mean of the three 2^-ΔΔCt^ values for each gene was calculated as the gene expression.

**Table 2:** The information of RT-qPCR primer sets used in this study.

Gene	Accession number	Primer sequences(5′-3′)	Product length (bp)	Amplification efficiency
*YWHAZ*	XM_006046432	F: GAAAGGGATTGTGGATCAG	184	2.012
		R: GGCTTCATCAAATGCTGTCT		
*GAPDH*	XM_006065800	F: ATGGAGAAGGCTGGGGCTCA	144	2.058
		R: GCAGGAGGCATTGCTGACAA		
*ACTB*	NM_001290932	F: TGGGCATGGAATCCTG	196	2.032
		R: GGCGCGATGATCTTGAT		
		R: ACCCTGGTCACAAACTGAATGCT		
*GPAM*	XM_006043939	F: ACTACGGATGTGTCAGAGTGGAT	141	1.986
		R:CACTGGGTCTTGAGGGAAGTATAG		
*DGAT2*	XM_006045187	F: GTCCTGTCTTTCCTCGTGCT	141	2.031
		R: CCTCCTGCCACCTTTCTT		
*AGPAT6*	NM_001290846	F:CTTTGCGTGGGCTACCTTG	242	2.0.32
		R: TCTTGGTCACCTCGTCGTC		
*BTN1A1*	NM_174508	F: GACTATCTGCCCAATCGCTGAT	153	2.116
		R: GGGCTGGAGAGGGATTAGTTTA		
*LPIN1*	XM_006073743	F: CAGTCGAGGCTCAGACCA	232	2.086
		R: TTCCCCGTTGATTTCTATGTCA		
*PPARG*	XM_006077448	F: GCTCCAAGAGTACCAAAGTG	204	2.096
		R: GTCCTCCTGAAGAAACCCTT		
*SREBF1*	KU517672	F: GCACCGAGGCCAAGTTGAATAA	146	2.041
		R: CAGGTCCTTCAGCGATTTGCTT		
*SREBF2*	XM_006068914	F:GCCAAGATGCACAAGTCTGGTGTT	136	2.099
		R: TGCCCTTCAGGAGCTTGCTCT		
*DGAT1*	NM_001290902	F: ACAGACAAGGACGGAGACG	268	1.988
		R: CCACAATGACCAGGCACA		

### TAG content assay

After the overexpression or knockdown of *LPIN1* for 48 h, the BMECs were rinsed twice with PBS. The TAG content in the BMECs was determined using the TAG kit (GPO-POD; Applygen Technologies Inc., Beijing, China) following the manufacturer’s instructions. Meanwhile, the intracellular total protein content was measured using the BCA protein assay kit (Thermo Fisher Scientific, USA). The TAG content was normalized by per milligram of protein.

### Cloning and bioinformatics analysis of buffalo LPIN1 promoter

Genomic DNA was extracted from the blood samples of Binglangjiang buffalo (adult, healthy) using the method of phenol/chloroform purification-based protocol. A pair of primers named LPIN1_F/R were designed to conduct the PCR for isolating the promoter of the *LPIN1* (Table 3). Amplified products were detected by 1.5% agarose gel electrophoresis. The target bands were excised from the agarose gel and then purified by Gel Extraction Kit (OMEGA, China). The purified PCR products were cloned into pMD18-T vector (TaKaRa, China) and further bidirectionally sequenced. Prediction of transcription factor binding sites in the promoter sequence was executed using online software JASPAR database (http://jaspar.genereg.net/) and Peroxisome Proliferator Response Elements Search (http://www.classicrus.com/PPRE/).

**Table 3:** Primers used in the construction of luciferase reporter vector.

Purpose	Primer ID	Primer sequences	Binding region (bp)	Anneal temperature/°C
Cloning	LPIN1_F	CTCGAGCCTAGGAGCCCAAGTCAGGT	− 1878	61.5
	LPIN1_R	*CCC*AAGCTTGCTGCCTTTGTATGCAAACACCC	+ 42	61.5
5’ deletion	F-1523	*CCG*CTCGAGTCAGACAGTGGAGGAGTCGTAA	− 1523	59.2
	F-1264	*CCG*CTCGAGATACTGGCGTGGGTAGCCGTTCC	− 1264	61.2
	F-953	*CCG*CTCGAGGTTAGTGACAGGGCTTGGAGTAT	− 953	59.4
	F-666	*CCG*CTCGAGCTGGGTGATTGCAGACCA	− 666	61.4
	F-416	*CCG*CTCGAGCTTCGCCAACGAGCACCAGA	− 416	62.9
	F-336	*CCG*CTCGAGACGGGCACTCTTGCTTCCTT	− 336	62.9
	F-172	*CCG*CTCGAGGGCTCACTCCTCGAAAGGGC	− 172	64.7
	F-88	*CCG*CTCGAGCTCTCGCACCACCCCGCTG	− 88	65
	R + 42	*CCC*AAGCTTGCTGCCTTTGTATGCAAACACCC	+ 42	61.5
Site-directed mutation	PPRE1	TGG**CC**TTG**TG**ACAG		62
	PPRE2	G**GG**TCGAAAGA**TC**T		62
	PPRE1-mut	CCCTGCAAAAGTCTGG**AG**TTG**AC**ACAGTAATAGGGAGAGG		59.2
	PPRE1-anti-mut	CCTCTCCCTATTACTGT**GT**CAA**CT**CCAGACTTTTGCAGGG		61.2
	PPRE2-mut	GCCCCGGGGGCGG**TA**TCGAAAGA**GT**TCTCGCACCAC		59.4
	PPRE2-anti-mut	GTGGTGCGAGA**AC**TCTTTCGA**TA**CCGCCCCCGGGGC		61.4

Plus ( +) and minus (-) indicate the number of nucleotides upstream (−) and downstream ( +) from the transcriptional start site. The underlined bases are the restriction site. The bases of italic are the protective bases. Mutation bases are shown in bold font.

### Construction of luciferase reporter gene vector

The complete buffalo *LPIN1* promoter fragment was excised from the pMD18-T vector with Xho I and Hind III restriction enzymes (TaKaRa, China), and then ligated into the pGL4.11 vector to generate the final promoter recombinant plasmid (pGL4-LPIN1). In addition, a series of upstream primers were designed using the complete promoter region sequence of *LPIN1* gene as a template, the following 5’ flanking deletion fragments of the promoter were further obtained by PCR: − 1523/ + 42, − 1264/ + 42, − 953/ + 42, − 666/ + 42, − 416/ + 42, − 336/ + 42, − 172/ + 42 and − 88/ + 42 (Table 3). The recombinant vectors of these 5’ flanking deleted promoter fragments were constructed, and all the fragments inserted into the vectors were confirmed by bidirectional sequencing. Then, the overlapping PCR method was used to generate site-directed mutagenesis with TIANSeq HIFI Amplification Mix (QIAGEN, Germany). The specific primers of overlapping PCR for site-directed mutagenesis promoters are shown in Table 3. All the PCR products were subsequently cloned into pGL4.11 vector, and all recombinant vectors were confirmed by DNA sequencing.

### Luciferase-based assays

When the confluence of BMECs in 12-well plates was 70–80%, the pGL4-LPIN1 vector or all recombinant vectors with 5’ deletion mutagenesis were co-transfected with pRL-TK vector in a ratio of 10:1 using Lipo6000™ transfection reagent (Beyotime Biotechnology). The pGL4.11 vector was co-transfected with pRL-TK as negative control. After 48 h of culture, the BMECs were collected for the determination of luciferase activity. The lentivirus (Lv-PPARG-sh3) used for *PPARG* gene knockdown and pEGFP-C1-PPARG used for overexpression of *PPARG* gene were constructed by our laboratory in the previous research^21^. In order to investigate the effect of *PPARG* overexpression and knockdown on the activity of the *LPIN1* promoter, after the BMECs were transfected with Lv-PPARG-sh3 or pEGFP-C1-PPARG for 12 h, the pGL4-LPIN1 plasmid and 5’deletion recombinant constructs of *LPIN1* promoter were transiently co-transfected the BMECs from the previous step with pRL-TK, and the BMECs were collected for RT-qPCR analysis and luciferase activity analysis after 48 h of culture. The BMECs were washed twice with cold PBS and luciferase activity was measured by Dual-Luciferase^®^ Reporter Assay System (Promega, Wisconsin, USA). The relative luciferase activity of each sample was normalized with pRL-TK.

### Statistical analysis

Three repetitions were set for all treatments, and the results were expressed as mean ± standard error of means (means ± SEM). Statistical comparisons (t-test) between the treatment and the control group were performed using software SPSS 19.0 (SPSS Inc., Chicago, IL) with a significance level of 0.05 and an extremely significant level of 0.01.

## Conclusion

In this study, in *vitro* overexpression and interference experiments showed that the interference with *LPIN1* significantly reduced the expression of lipid synthesis related genes and TAG content in the BMECs, while the overexpression of *LPIN1* significantly increased the expression of lipid synthesis related genes and TAG content in the BMECs. Further transcriptional regulation experiments showed that the overexpression or interference of *PPARG* could enhance or decrease the expression and promoter activity of buffalo *LPIN1*, and the site-directed mutagenesis of PPRE1 and PPRE2 significantly reduced the promoter activity of buffalo *LPIN1*. The results here suggest that buffalo LPIN1 is involved in the synthesis of milk fat, and the PPARG can directly regulate the transcription of buffalo *LPIN1* by binding to the PPRE1 and PPRE2 in its core promoter.

## Data Availability

The data analyzed during the current study are available from the corresponding author on reasonable request.
